# Circulating Autoantibodies Against Vasoactive Biomarkers Related to Orthostatic Intolerance in Long COVID Patients Compared to No-Long-COVID Populations: A Case-Control Study

**DOI:** 10.3390/biom15020300

**Published:** 2025-02-18

**Authors:** Emilie Han, Katrin Müller-Zlabinger, Ena Hasimbegovic, Laura Poschenreithner, Nina Kastner, Babette Maleiner, Kevin Hamzaraj, Andreas Spannbauer, Martin Riesenhuber, Anja Vavrikova, Antonia Domanig, Christian Nitsche, Dominika Lukovic, Thomas A. Zelniker, Mariann Gyöngyösi

**Affiliations:** Division of Cardiology, Department of Internal Medicine II, Medical University of Vienna, 1090 Vienna, Austriathomas.zelniker@meduniwien.ac.at (T.A.Z.)

**Keywords:** long COVID, post COVID, autoantibodies, vasoactive peptides, orthostatic intolerance, endothelial dysfunction

## Abstract

Endothelial dysfunction mediated by elevated levels of autoantibodies against vasoactive peptides occurring after COVID-19 infection is proposed as a possible pathomechanism for orthostatic intolerance in long COVID patients. This case-control study comprised 100 long COVID patients from our prospective POSTCOV registry and three control groups, each consisting of 20 individuals (Asymptomatic post-COVID group; Healthy group = pan-negative for antispike protein of SARS-CoV-2; Vaccinated healthy group = no history of COVID-19 and vaccinated). Autoantibodies towards muscarinic acetylcholine receptor M3, endothelin type A receptor (ETAR), beta-2 adrenergic receptor (Beta-2 AR), angiotensin II receptor 1 and angiotensin 1-7 (Ang1-7) concentrations were measured by enzyme-linked immunosorbent assay in long COVID patients and controls. Orthostatic intolerance was defined as inappropriate sinus tachycardia, postural tachycardia, orthostatic hypotonia and other dysautonomia symptoms, such as dizziness or blurred vision (*n* = 38 long COVID patients). Autoantibody concentrations were compared with routine laboratory parameters and quality of life questionnaires (EQ-5D). The concentration of ETAR autoantibodies were significantly higher in long COVID, Asymptomatic and Vaccinated groups compared to the antispike protein pan-negative Healthy group. A trend towards higher plasma levels of Beta-2 AR and Ang1-7 was measured in long COVID patients, not related to presence of orthostatic intolerance. ETAR autoantibody concentration showed significant positive correlation with the EQ-5D item “Problems in performing usual activities”.

## 1. Introduction

The pathophysiology of orthostatic intolerance, postural orthostatic tachycardia syndrome (POTS) and post-exertional malaise (PEM) as well as many other cardiovascular symptoms in long COVID patients is still poorly understood. Hypotheses regarding the underlying mechanisms are mainly based on mitochondrial dysfunction [1], cellular oxygen deprivation or endothelial dysfunction [2,3]. Long COVID can involve multiple organ systems and present with either a single or several main symptoms, or a wide array of unspecific health problems [4]. This heterogenicity in patients’ presentation coupled with sparse biochemical objective markers are the main challenges in diagnosing this new post-viral illness [5,6].

Recently, new insights were gained on the pathomechanisms of PEM in long COVID patients, where a reduction in skeletal muscle mitochondrial enzyme activity and an increased accumulation of amyloid-containing deposits in skeletal muscle compared to controls were discovered to causing the symptoms [7]. However, these data do not support the hypothesis of tissue hypoxia caused by blockage of small blood vessels as a possible pathophysiological cause of long COVID [7]. Additionally, the possibility of endothelial dysfunction as a primary contributor of long COVID symptoms seems promising, since problems in vasculature can affect the blood vessels in the brain, lung, heart or in the periphery [8].

Vasoactive peptides are responsible for regulation of blood pressure through the renin-angiotensin aldosterone system and sympathetic nervous system [9]. Many of those factors are part of the G protein-coupled receptors (GPCR) signaling pathways, such as the β2 adrenoreceptor, α1 adrenoreceptor or angiotensin II receptor 1 (ATR1). In long COVID patients autoantibodies targeting these receptors have been found to possess implication in postural orthostatic tachycardia syndrome (POTS) [10]. Recently, a study with prospectively enrolled hospitalized COVID patients found higher concentrations of auto-antibodies against ATR1 and endothelin receptor type A (ETAR) compared to controls [11]. ETAR belongs to the group of G-protein coupled receptors, which are activated by endothelin-1 to facilitate vasoconstriction, while autoantibodies against ETAR can act as functional agonists [12]. Similarly, muscarinic acetylcholine receptor 3 (mAChR3) has an important role in the cardiovascular system by parasympathetic signaling pathways inducing endothelium-dependent vasodilation of the coronary arteries or modulating heart rate and cardiomyocyte repolarization, thereby regulating vascular tone and cardiac dynamics [13], which makes it an interesting target to investigate in post-COVID individuals with orthostatic symptoms. Additionally, the alternative renin-angiotensin-system (RAS) pathway consisting of the ACE2/Ang1-7/MAS axis, with cardioprotective and anti-inflammatory effects, has been of interest and studied in acute COVID-19, although with inconclusive results, but not in long COVID yet [14].

Furthermore, another paper reported correlation of neurological symptom severity with autoantibodies targeting vasoregulatory and autonomic nervous system receptors in long COVID patients, compared to non-infected and post-COVID individuals [15]. Similarly, autoantibodies against beta-2 adrenergic receptor (Beta-2 AR) were found to correlate with symptom severity in post-COVID patients [16]. However, although macrovascular dysfunction was not found in the majority long COVID patients, evidence suggests endothelial dysfunction playing a key role caused by increased activation of pro-coagulatory factors and elevated endothelin-1 levels in some of post-COVID individuals compared to non-infected controls [17].

The aim of this study was to measure autoantibody concentrations of vasoactive peptides in the sera of long COVID patients and assess possible association with symptom burden. This may further reveal possible pathomechanisms to the multi-faceted problem of determining the risk factors of long COVID focusing on orthostatic intolerance and other cardiovascular symptoms.

## 2. Materials and Methods

### 2.1. Ethics Statement

The long COVID patients from this study were recruited as part of the ongoing POSTCOV prospective registry, approved by the Ethical Committee of the Medical University of Vienna (EC: 1008/2021 approval date: 24 February 2021, and EC: 1758/2022 approval date: 11 November 2022, ClinicalTrials.gov Identifier: NCT05398952). This study was performed in accordance with the Declaration of Helsinki. The control cases with no long COVID were selected from a separate cohort (EC: 1387/2020 approval date: 20 April 2020). This research adheres to ethical regulations regarding clinical work with humans, and written informed consent was obtained from all participating patients. Reporting follows the STROBE checklist [18].

### 2.2. Study Population

Inclusion criteria for long COVID patients consisted of real-time PCR-confirmed SARS-CoV-2 infection with mild to moderate COVID-19 during the acute phase (not requiring hospitalization); extensive screening investigations were used to exclude other possible organ diseases causing the ongoing symptoms before the clinical diagnosis of Long-COVID syndrome was made. Exclusion criteria for the long COVID cohort comprised a history or current systemic inflammatory diseases or active malignancies. Long COVID patients were recruited between March 2021 and March 2022. “Asymptomatic” controls were individuals after recovered COVID-19 infection (meaning no long COVID symptoms) recruited between June 2021 and October 2021, “Vaccinated” controls were individuals who received COVID-19 vaccination recruited between October 2021 and February 2022, but were not infected (negative nucleocapsid antigen), while “Healthy” controls were completely naïve towards the COVID-19 antispike protein as well as negative for nucleocapsid antigen and had no previous symptoms of a coronavirus infection, recruited from May 2020 to June 2020. Individuals in the “Vaccinated” group were selected based on self-reported vaccination status and medical health records including vaccination status and date. The samples for the “Healthy” control group were collected early in the pandemic from healthcare workers who underwent frequent and regular COVID-19 testing (2–3 times/week, minimum once weekly) as part of their routine hospital duties. With these three control groups a comprehensive comparison of long COVID patients with different previous COVID-19 or vaccination conditions could be accomplished. Blood samples were retrieved from the Biobank of the Medical University of Vienna.

### 2.3. Clinical Data

Clinical data were assessed at the time of the first clinical presentation in the long COVID outpatient clinic and if needed at subsequent follow-up visits. Patient history, comorbidities, pre-existing cardiovascular and pulmonary conditions, date of COVID infection(s), vaccination status and date(s) of vaccination, previous and current medications, quality of life surveys, vital signs (blood pressure, heart rate), ECG and findings from preceding diagnostic procedures (i.e., chest X-ray, spirometry, Holter-ECG, echocardiography, exercise testing) were recorded at each study visit. Additional tests, such as computed tomography, cardiac magnetic resonance imaging or neurological investigations, were ordered to rule out any other objective and other possible well-defined organ diseases causing the symptoms. Orthostatic intolerance was defined as inappropriate sinus tachycardia, postural tachycardia, orthostatic hypotonia and other dysautonomia symptoms such as dizziness or blurred vision.

Quality of life was assessed with the EQ-5D-3L questionnaire in German including a visual analogue scale (VAS) for general quality of life (range 0–100) [19]. An additional questionnaire measured different symptom burdens at baseline visit and before COVID-19 infection by self-assessment based on the COVID-19 Yorkshire Rehabilitation Scale (C19-YRS) [20].

The presence of following comorbidities was recorded according to the following definitions: hypertension (any blood pressure lowering medication or blood pressure at baseline above 140/90 mmHg), diabetes (any antidiabetic medication or insulin usage, or HbA1c >6.4%), hyperlipidemia (any lipid-lowering medication or cholesterol >200 mg/dL) and current smoking by self-assessment.

### 2.4. Laboratory Data

All routine laboratory assessments including blood levels of antibodies against SARS-CoV-2 were performed at the Department of Laboratory Medicine of the Medical University of Vienna. The quantitative amounts of circulating antispike protein antibodies were measured by cobas^®^ e 801 analyzer (Roche Diagnostics, Rotkreuz, Switzerland) with the Elecsys Anti-SARS-CoV-2 S electro-chemiluminescence immunoassay (Roche Diagnostics) (range: 0.4–2500.0 U/mL, with 0.8 U/mL as a cutoff for positivity) and was performed in accordance with the manufacturer’s instructions. Sandwich enzyme-linked immunosorbent assay (ELISA) kits were used for the measurement of autoantibodies towards endothelin receptor type A (ETAR) (CellTrend GmbH, REF 12100, Luckenwalde, Germany), beta-2 adrenergic receptor (CellTrend GmbH, REF 12700, Luckenwalde, Germany), muscarinic acetylcholine receptor M3 (CellTrend GmbH, REF 15300, Luckenwalde, Germany) and human angiotensin II receptor 1 (ATR1) (AssayGenie, HUFI02205, Dublin, Ireland). A competitive ELISA kit was used to measure angiotensin 1-7 (Ang1-7) concentration (Cloud-Clone Corp, CES085Hu, Lot Nb: L231203652, Katy, TX, USA). Only reagents from the corresponding kits were used. Pre-tests were performed to determine the optimum dilution factor for the samples. ELISA were performed with serum samples retrieved from our biobank. An automated ELISA plate washer (Thermo Scientific™ Wellwash™ Microplate Washer, Thermo Fisher Scientific, Madrid, Spain) was used and a microplate reader (Ao Absorbance Microplate ReaderAC3000, Azure Biosystems, Dublin, CA, USA) with the optical density absorbance set at 450 nm for all reactions, as specified by the kits’ workflow protocols. Analysis for concentration assessment was performed using a four parameter logistic (4PL) curve, with calibrators set as specified by each ELISA kit, on the webtool Assayfit Pro (https://www.assayfit.com, last accessed on 20 March 2024).

### 2.5. Statistical Analysis

Categorical data were shown as counts and percentages, while continuous data were described as median and interquartile range (IQR) for descriptive statistics. Differences between the different study groups were assessed using ANOVA and the Kruskal–Wallis test. Correlation analyses were computed as nonparametric Spearman correlations (r). Missing data was shown in the tables of descriptive statistics. For the correlation analysis of questionnaire data with ETAR autoantibodies a complete case analysis approach was used. *p* values were adjusted for multiple testing with the Dunn’s test. A two-tailed *p* value ≤ 0.05 was considered statistically significant. The statistical program used for analysis and visualization was Prism (Version 10.4.0). 

## 3. Results

### 3.1. Study Population Characteristics

Overall, one hundred long COVID patients were included in this study, while the three control groups, healthy controls (unvaccinated and non-infected, e.g., pan-negative for antispike protein), vaccinated healthy (=Vaccinated without SARS-CoV-2 infection) and infected-no long COVID (=Asymptomatic) each consisted of twenty participants (Table S1). Seventy-four percent of the study participants were female. The mean age (43.8 ± 12.4 years) was comparable between the groups with the exception of the vaccinated controls (52.7 ± 7.1 years).

### 3.2. Circulating Autoantibodies Against Vasoactive Peptides

**Figure 1:**
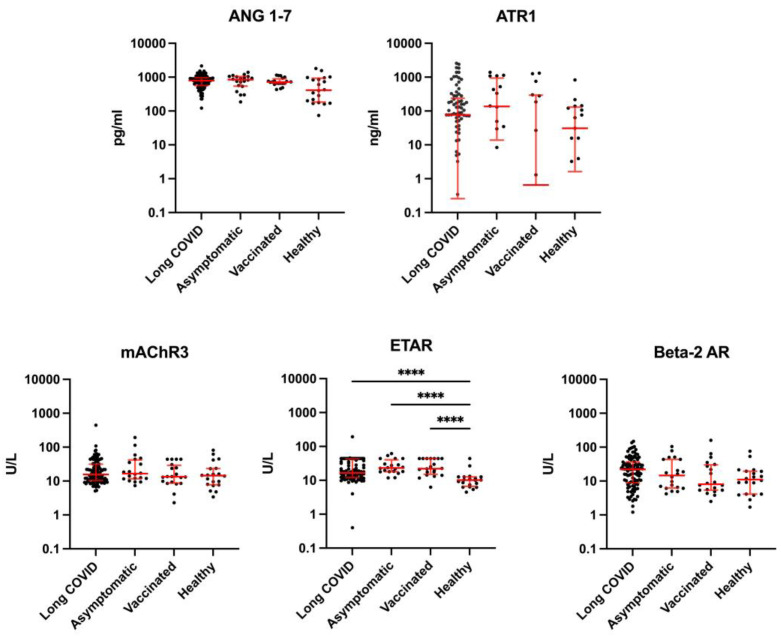
Concentration of Ang1-7 and levels of antibodies against Angiotensin II receptor 1 (ATR1), muscarinic acetylcholine receptor 3 (mAChR3), endothelin A receptor (ETAR) and beta-2 adrenergic receptor (Beta-2 AR) in long COVID patients and the 3 control groups (Asymptomatic = infected with no long COVID, Healthy = unvaccinated and non-infected, Vaccinated = vaccinated and non-infected). Median and IQR (shown as error bars), **** indicates significant difference (*p* values < 0.0001) between groups. (Additional enlarged image of ETAR panel can be found in the Supplementary Materials: Supplementary Figure S1).

Endothelin receptor type A autoantibodies were increased in study groups who had contact with SARS-CoV-2 spike protein (*p* < 0.0001, ANOVA). The concentration of autoantibodies towards endothelin receptor type A (ETAR) were significantly higher in the blood sera of long COVID, Asymptomatic and Vaccinated groups compared to those of the pan-negative Healthy group (Figure 1). No differences in autoantibody concentrations could be observed for mAChR3 or ATR1, while antibodies towards Beta-2 AR showed a higher trend for long COVID patients compared to the Healthy and Vaccinated control groups (*p* = 0.0634, ANOVA). Similarly, concentration of Ang1-7 was not different between long COVID patients and control groups, though a trend of higher values could be observed for long COVID patients compared to the Healthy group (*p* = 0.0741, ANOVA) (Figure 1).

There were several significant but weak linear correlations between measured autoantibodies against vasoactive peptides (Supplementary Figure S2A). ETAR autoantibody concentrations possessed significant correlations with all other auto-antibodies measured, with moderate positive correlations for mAChR3 (r = 0.51, 95% CI: 0.38 to 0.62, *p* < 0.0001) and ATR1 (r = 0.28, 95% CI: 0.13 to 0.43, *p* = 0.0002), but weak correlation with Ang1-7 (r = 0.20, 95% CI: 0.04 to 0.35, *p* = 0.0125) (Figure S2B–E). The weak correlation between the measured vasoactive peptides mirrors the association between the neurohumoral and vasoactive peptides.

### 3.3. Characterization of Long COVID Patients With or Without Orthostatic Intolerance

#### 3.3.1. Clinical and Laboratory Data

Thirty-eight long COVID patients (68.4% female) had orthostatic intolerance with a median age of 38 (IQR: 33.0–48.2). The autoantibody concentrations against vasoactive peptides did not differ between long COVID patients with orthostatic intolerance (i.e., median ETAR 16.4 U/L, IQR: 12.3–22.6) compared to those without (median ETAR 16.8 U/L, IQR: 12.3–44.0; *p* = 0.174) (Table 1). Median antispike protein concentration in long COVID patients with orthostatic intolerance was 1267.5 BAU/mL [IQR: 78.9–2500.0], while it was 2500.0 BAU/mL [IQR: 105.8- 2500.0; *p* = 0.668] in patients without orthostatic intolerance. There was no correlation between ETAR autoantibody concentration and antispike protein concentration (r = 0.013, *p* = 0.896) (Supplementary Figure S3).

Detailed patient characteristics of the hundred long COVID patients included in this analysis stratified by orthostatic intolerance and vaccination status are displayed in Table 2. Vital parameters, such as blood pressure and heart rate did not differ between the groups. General quality of life measure via a visual analogue scale did not differ between groups either. Long COVID patients who were unvaccinated before the baseline visit presented sooner at our outpatient clinic after COVID-19 positivity irrespective of orthostatic intolerance (i.e., orthostatic intolerance and unvaccinated: 182.50 days, IQR: 107.00–256.00, vs., no orthostatic intolerance and unvaccinated: 264.00 days, IQR: 186.25, 332.50). Routine laboratory parameters only had weak or very weak correlation with the autoantibody concentrations against vasoactive peptides (Supplementary Figure S4).

Additionally, most of the baseline characteristics were comparable between female and male long COVID patients (Supplementry Table S2). Notable differences were present for IgM (F: 109.0 mg/dL, IQR: 81.1–161.0 vs. M: 75.4 mg/dL, IQR: 63.5–115.0) and IgE (F: 37.0 kIU/L, IQR: 20.4–78.0 vs. M: 7.6 kIU/L, IQR: 4.7–34.0) concentrations, which were significantly higher in women compared to men (Table S2).

#### 3.3.2. Prevalence of Long COVID Symptoms

The most commonly reported symptoms overall were fatigue, difficulty concentrating and sleep problems. In female patients, fatigue (59.5%), dyspnea/shortness of breath (41.9%) and concentration problems (36.5%) were listed as most commonly reported symptoms, while in male patients they were concentration problems (53.8%), dyspnea/shortness of breath (46.2%), and cardiac symptoms (palpitations and chest pain) (42.3%).

In long COVID patients with orthostatic intolerance, which was defined as sinus tachycardia, postural tachycardia, orthostatic hypotonia and other dysautonomia symptoms, the following symptoms were present: 71% of them had sinus tachycardia and/or palpitations. Additionally, 50% complained of chest pain/discomfort. Brain fog and fatigue were prevalent in 34% and 42% of orthostatic intolerant long COVID patients, respectively. Lastly, the symptoms of headache and dizziness could be assessed in 58% and PEM in 61% of patients.

### 3.4. ETAR Autoantibody Concentration Positively Correlates with Greater Difficulty Performing Usual Activities in Long COVID Patients

Fully completed EQ-5D-3L questionnaires were available for 60 long COVID patients at the baseline visit. Long COVID patients had the least amount of problems in self-care activities, but the most commonly reported was having some problems in doing usual activities, such as working, studying, housework or doing activities in leisure time (Table 3). None of the patients reported not being able to walk around at all. Some level of pain or discomfort (up to extreme level) was present in almost all with available questionnaires (55/60). ETAR autoantibody concentration showed significant positive correlation with the EQ-5D item “Usual activities” (r = 0.33, *p* = 0.0099), irrespective from having orthostatic intolerance (Table 4). Other autoantibodies against vasoactive peptides did not possess any significant correlation with EQ-5D items.

## 4. Discussion

Autoantibody concentration of ETAR was elevated in patients with circulating antispike protein, which might explain endothelial dysfunction. However, none of the investigated vasoactive peptides had an absolute discriminatory function between Long-COVID syndrome and healthy vaccinated or pan-negative controls and asymptomatic post-COVID patients. A strong trend (*p* < 0.1) towards elevated Beta-2 AR and Ang1-7 was observed in long COVID patients, however, irrespective from the presence of orthostatic intolerance. In long COVID patients elevated autoantibodies against ETAR correlated with more symptoms in performing usual daily activities. Thirty-eight of a hundred long COVID patients from our center had orthostatic intolerance, while unvaccinated persons presented earlier to our outpatient clinic after COVID-19 infection compared to vaccinated ones.

Orthostatic intolerance emerging post-COVID-19 has been attributed to possible dysfunction in inflammatory and autoimmunity pathways, changes in signaling along the neuro-cardiac axis or hypovolemia mediated by renin-angiotensin-aldosterone system (RAAS) disbalance [21]. The RAAS imbalance may also be involved in the pathophysiology of POTS by potentially amplifying inflammatory and sympathetic activation following ACE-2 receptor downregulation caused by the virus as an autoimmune mechanism [22]. Other authors found inappropriate sinus tachycardia in 20% of individuals with post-COVID-19 without underlying structural heart disease, myocyte injury, or proinflammatory state, which may be related to cardiac autonomic nervous system imbalance [23]. Notably, a numeric difference was observed, with a shorter duration since receiving the COVID-19 vaccine to baseline presentation in long COVID patients with orthostatic intolerance versus no orthostatic intolerance (seen in Table 1). Further, when stratified by vaccination status, the vaccinated long COVID patients took longer to present for the baseline visit after COVID-19 infection irrespective of presence or absence of orthostatic intolerance (as seen in Table 2). This could be attributed to the finding that COVID-19 vaccination reduces the risk of long COVID, regardless of when the vaccination occurs in relation to the COVID-19 infection, as seen from a meta-analysis of over one million patients across 18 studies [24].

Elevated concentrations of autoantibodies against G-coupled protein receptors against vasoactive peptides have been found in association with acute COVID-19 and in post-COVID patients before. Similar to our results, several autoantibody concentrations measured by ELISA were found to differ between long COVID and no long COVID patient groups [15]. While they have mainly investigated neurologic symptoms of long COVID in regard to these autoantibodies, our analysis focused on orthostatic intolerance and cardiovascular symptoms. Cardiovascular neurohormones, such as epinephrine, vasopressin and pro-adrenomedullin, have been found to affect timely onset of vasovagal syncope in a head-up tilt test through regulation of the autonomic nervous system [25]. A previous study looked into gene variant encoding for endothelin-1 and ETAR and identified an endothelin-1 with a potential role in the pathophysiology of vasovagal syncope, which, like orthostatic intolerance, is characterized by a dysregulated autonomic nervous system [26]. Numerically lower concentrations of mAChR3 autoantibodies were found in the orthostatic intolerance group. However, the difference was not statistically significant as compared to the group without orthostatic intolerance. Additionally, according to the kit’s manufacturer instructions, the categorical cut-off value lies at >10 U/L, which is regarded as positive, thus the median values of 12.8 vs. 20.1 U/L in the groups do not have a relevant implication.

In previously published work, we have shown that anti-inflammatory medication and condition-adapted heart failure treatment using ACEi or ARB and betablockers improved long COVID symptoms and decreased cardiac abnormalities found in magnetic resonance imaging [27]. Thus, it could provide a viable option to target inflammation and the RAAS for long COVID treatment to improve cardiovascular complaints. At the time of publication, results of a phase II study on the safety and efficacy of daily doses of celecoxib and valacyclovir in long COVID patients have yet to be published [28]. Unfortunately, no randomized control trials investigating the effectiveness of RAAS inhibitors in long COVID patients have been conducted to date, representing a significant gap in the current body of evidence, despite several established hypothesis in literature linking the RAAS with the pathophysiology of long COVID [29].

Immunological dysregulation may be one of the main culprits in many long COVID symptoms. A longitudinal study looked into different T cell activation and subset patterns in patients with Long-COVID syndrome [30]. They found that cellular mechanisms of long COVID were dependent on initial disease severity. Individuals who have suffered from only mild acute disease showing more naïve and decreased central memory and effector memory CD4+ Treg subsets [30]. Furthermore, circulating inflammatory biomarkers seem also to play a role in the disease course of long COVID, as a combination of IFN-β, PTX3, IFN-γ, IFN-λ2/3 and IL-6 were found to be 78.5–81.6 accurate for association with long COVID [31]. The elevated autoantibody levels against vasoactive peptides mirrors also the false immunological reactions caused by the SARS-CoV-2 infection, and that individual autoimmunity risk may impact the disease development and progression of long COVID.

Thus, immunomodulatory agents, such as therapies with steroids (systemic or inhalation) or intravenous immunoglobulins (IVIG) in currently ongoing trials are undergoing investigation as potential treatment options for long COVID patients [32,33]. Furthermore, non-pharmacological treatments, such as physical rehabilitation programs (daily inspiratory muscle training combined with aerobic and resistance exercises 3 times per week) have shown improvement in cardiorespiratory fitness and decrease of symptom burden in long COVID patients [34].

### Limitations

One of this study’s limitation encompasses the number of patients included in the analysis; investigations in a larger cohort are needed to further confirm our findings. Another aspect is the potential for underdiagnosed orthostatic intolerance due to difficulties in diagnostic procedures in routine clinical care and unspecific presenting symptoms. However, in this closely followed-up cohort of ours, referrals to other departments at our center, such as neurology, angiology or pneumology, were available to pursue further testing to dismiss other possible disease etiologies. Additionally, future studies may include extended follow-up periods with longitudinal measurements of these autoantibody levels in long COVID patients, to better understand their long-term implications on symptom progression and its impact on comorbidities as well as the possible effects of concurrent medications. According to our results, that show no significant correlation between ETAR and antispike protein, we can exclude a direct cross-reactivity between these two biomarkers. In vitro testing of cross-reactivity with recombinant spike protein and ETAR would be helpful; however, this experiment was beyond the scope of this study, but future research should address this to clarify antibody specificity.

## 5. Conclusions

ETAR autoantibodies were found to be different between individuals with long COVID, no long COVID, or vaccination previously and completely COVID-spike naïve people. A trend towards elevated Beta-2 AR and Ang1-7 was observed in long COVID patients, although irrespective from presence of orthostatic intolerance. Additionally, ETAR autoantibody concentrations correlate with more reported problems with usual daily activities. In general, symptom burden was moderate, at about 50% from the maximum 100% of general quality of life. In conclusion, our findings suggest that ETAR autoantibodies may be a potential biomarker of vascular or autonomic dysfunction in long COVID patients. However, further research is needed to determine their specificity and establish their diagnostic or prognostic value for long COVID and other post-viral syndromes.

## Figures and Tables

**Table 1:** Baseline characteristics of long COVID patients with and without orthostatic intolerance.

	Orthostatic Intolerance Group (*n* = 38)	No Orthostatic Intolerance (*n* = 62)	*p* Value
Age (year)	38.0 [33.0, 48.2]	37.5 [32.0, 53.8]	0.301
Sex female	26 (68.4%)	48 (77.4%)	0.319
BMI (kg / m^2^ )	22.8 [20.5, 26.1] (*n* = 30)	24.4 [21.7, 26.9] (*n* = 47)	0.477
Hypertension	9 (23.7%)	21 (33.9%)	0.281
Diabetes mellitus	0 (0%)	0 (0%)	-
Hyperlipidemia	6 (15.8%)	12 (19.4%)	0.652
Smoking (current)	6 (22.2%) (*n* = 27)	6 (13.3%) (*n* = 45)	0.327
COVID-19 vaccinated at baseline	16 (42.1%)	32 (51.6%)	0.356
SpikeAB (BAU/mL)	1267.5 [78.9, 2500.0]	2500.0 [105.8, 2500.0]	0.668
Time between COVID vaccine and baseline visit (days)	22.5 [1.2, 97.8]	70.5 [14.5, 143.2]	0.597
Time between COVID-19 positivity and baseline visit (days)	231.0 [155.0, 338.8]	219.5 [165.2, 298.2]	0.544
Systolic blood pressure (mmHg)	130.0 [120.0, 135.0] (*n* = 37)	130.0 [120.0, 140.0] (*n* = 55)	0.379
Diastolic blood pressure (mmHg)	85.0 [75.0, 90.0] (*n* = 37)	85.0 [80.0, 90.0] (*n* = 55)	0.448
CONCOMITANT MED			
Betablockers	3 (7.9%)	7 (11.3%)	0.583
RAS inhibitors			0.098
None	34 (89.5%)	54 (87.1%)	
ACE inhibitors	2 (5.3%)	0 (0.0%)	
ARB	2 (5.3%)	8 (12.9%)	
Lipid lowering medication	3 (7.9%)	6 (9.7%)	0.762
AUTOANTIBODIES AGAINST VASOACTIVE PEPTIDES			
mAChR3 (U/L)	12.8 [9.7, 23.5]	20.1 [11.6, 33.1]	0.310
ETAR (U/L)	16.4 [12.3, 22.6]	16.8 [12.3, 44.0]	0.174
Beta-2 AR (U/L)	12.5 [7.6, 16.6]	14.4 [10.4, 29.2]	0.199
Ang1-7 (pg/mL)	801.6 [547.0, 915.2]	790.6 [588.6, 994.5]	0.339
ATR1 (ng/mL)	113.0 [19.0, 927.6]	144.6 [7.0, 1032.0]	0.955
ROUTINE LABORATORY PARAMETERS			
Hemoglobin (g/dL)	14.2 [13.4, 14.7] (*n* = 37)	13.6 [12.9, 14.6] (*n* = 59)	0.207
Hematocrit (%)	41.8 [40.1, 44.4] (*n* = 37)	41.2 [39.8, 43.5] (*n* = 59)	0.554
MCV (fl)	89.7 [87.7, 90.4] (*n* = 37)	89.8 [87.7, 91.4] (*n* = 59)	0.698
MCH (pg)	30.0 [29.3, 30.6] (*n* = 37)	29.9 [28.9, 30.6] (*n* = 59)	0.465
Platelets (G/L)	264.0 [229.0, 292.0] (*n* = 37)	248.0 [214.0, 288.0] (*n* = 59)	0.810
Leucocytes (G/L)	6.5 [5.5, 7.6] (*n* = 37)	6.2 [5.5, 6.9] (*n* = 59)	**0.031**
NT-proBNP (pg/mL)	44.8 [31.7, 66.5] (*n* = 37)	51.5 [30.4, 83.6] (*n* = 59)	0.321
hs-CRP (mg/dL)	0.1 [0.0, 0.2] (*n* = 35)	0.1 [0.1, 0.2] (*n* = 56)	0.498
Interleukin-6 (pg/mL)	1.5 [1.5, 2.1] (*n* = 37)	1.6 [1.5, 2.6] (*n* = 59)	0.562
IgG (mg/dL)	1140.0 [1000.0, 1280.0] (*n* = 37)	1070.0 [970.0, 1220.0] (*n* = 61)	0.251
IgA (mg/dL)	196.0 [151.0, 282.0] (*n* = 37)	182.0 [138.0, 226.0] (*n* = 61)	0.071
IgM (mg/dL)	102.0 [65.9, 146.0] (*n* = 37)	102.5 [75.3, 140.0] (*n* = 58)	0.673
IgGsubclass1 (mg/dL)	699.0 [615.2, 796.8] (*n* = 34)	679.0 [586.5, 783.0] (*n* = 59)	0.576
IgGsubclass2 (mg/dL)	329.0 [265.8, 424.8] (*n* = 34)	335.0 [264.5, 420.0] (*n* = 59)	0.784
IgGsubclass3 (mg/dL)	34.0 [23.2, 41.2] (*n* = 34)	33.2 [24.4, 43.2] (*n* = 59)	0.636
IgGsubclass4 (mg/dL)	53.8 [22.2, 83.8] (*n* = 34)	40.9 [23.8, 96.7] (*n* = 59)	0.629
IgE (kIU/L)	37.4 [15.6, 79.7] (*n* = 36)	25.9 [9.3, 47.2] (*n* = 58)	0.182
ECHO PARAMETERS			
Left ventricle size (mm)	43.0 [40.0, 47.0] (*n* = 27)	42.0 [40.0, 45.0] (*n* = 41)	0.611
Right ventricle size (mm)	29.0 [26.0, 32.2] (*n* = 28)	28.0 [26.0, 31.0] (*n* = 41)	0.224
Right atrium size (mm)	44.0 [42.2, 49.5] (*n* = 26)	45.0 [40.0, 48.0] (*n* = 41)	0.734

Continuous data displayed as median [Q1, Q3]. Categorical data displayed as counts (*n*) and column percentages (%). *p* value for comparison between orthostatic intolerance group and no orthostatic intolerance. ACEi = angiotensin converting enzyme inhibitor, Ang1-7 = Angiotensin (1-7), ARB = angiotensin receptor blocker, ATR1 = Angiotensin II receptor 1, BAU = binding antibody units, Beta-2 AR = Beta-2 adrenergic receptor, BMI = body mass index, ETAR = endothelin receptor type A, hs-CRP = high-sensitivity C-reactive protein, mAChR3 = muscarinic acetylcholine receptor 3, SpikeAB = Spike Protein Antibody. Bold numbers indicate significant *p* Values.

**Table 2:** Long COVID patients with and without orthostatic intolerance stratified into vaccinated and non-vaccinated subgroups.

	Orthostatic Intolerance *n* = 38		No Orthostatic Intolerance *n* = 62		
Characteristic	Orthostatic Intolerance & Unvaccinated *n* = 22 ^1^	Orthostatic Intolerance & Vaccinated *n* = 16 ^1^	No Orthostatic Intolerance & Unvaccinated *n* = 30 ^1^	No Orthostatic Intolerance & Vaccinated *n* = 32 ^1^	*p*-Value ^2^
Age (year)	37.00 [33.00, 48.25]	40.00 [32.00, 46.75]	37.50 [33.00, 53.75]	37.50 [31.00, 53.50]	0.950
Female	16 (73%)	10 (63%)	25 (83%)	23 (72%)	0.466
BMI (kg / m^2^ )	21.25 [20.30, 25.19]	24.02 [21.60, 26.05]	23.08 [19.84, 25.65]	24.49 [22.76, 27.70]	0.193
Systolic blood pressure (mmHg)	130.00 [115.00, 140.00]	127.50 [123.75, 130.00]	130.00 [120.00, 145.00]	130.00 [120.00, 140.00]	0.944
Diastolic blood pressure (mmHg)	80.00 [75.00, 90.00]	85.00 [78.75, 90.00]	85.00 [80.00, 90.00]	85.00 [80.00, 90.00]	0.909
Heart rate (bpm)	75.00 [65.00, 80.00]	71.00 [61.75, 79.75]	70.50 [64.75, 74.00]	72.00 [67.50, 82.00]	0.513
Hypertension	4 (18%)	5 (31%)	9 (30%)	12 (38%)	0.506
Diabetes mellitus	0 (0%)	0 (0%)	0 (0%)	0 (0%)	-
Hyperlipidemia	2 (9.1%)	4 (25%)	4 (13%)	8 (25%)	0.359
Smoking (current)	2 (14%)	4 (31%)	2 (10%)	4 (16%)	0.463
Visual analogue scale of general quality of life (range 0–100)	55.00 [40.00, 65.00]	62.50 [39.25, 68.75]	60.00 [41.25, 65.00]	40.00 [34.00, 65.00]	0.612
Time between COVID vaccine and baseline visit (days)	-	75.00 [23.25, 147.00]	-	108.00 [57.50, 154.50]	**<0.001**
Time between COVID-19 positivity and baseline visit (days)	182.50 [107.00, 256.00]	269.00 [220.75, 356.25]	194.50 [140.50, 234.50]	264.00 [186.25, 332.50]	**0.022**
CONCOMITANT MEDICATION					
Betablockers	1 (4.5%)	2 (13%)	2 (6.7%)	5 (16%)	0.506
RAS inhibitors					0.497
None	20 (91%)	14 (88%)	27 (90%)	27 (84%)	
ACE inhibitors	1 (4.5%)	1 (6.3%)	0 (0%)	0 (0%)	
ARB	1 (4.5%)	1 (6.3%)	3 (10%)	5 (16%)	
Lipid lowering medication	0 (0%)	3 (19%)	0 (0%)	6 (19%)	**0.013**
AUTOANTIBODIES AGAINST VASOACTIVE PEPTIDES					
mAChR3 (U/L)	14.58 [9.63, 28.05]	12.46 [10.37, 15.03]	17.13 [11.60, 32.13]	20.76 [11.74, 39.13]	0.294
ETAR (U/L)	16.75 [12.70, 21.43]	16.41 [12.96, 28.99]	15.82 [11.50, 40.08]	24.79 [13.49, 44.00]	0.345
Beta-2 AR (U/L)	12.50 [7.97, 18.33]	12.50 [8.27, 15.89]	13.80 [10.91, 28.56]	15.05 [9.21, 29.10]	0.521
Ang1-7 (pg/mL)	872.35 [708.74, 997.10]	623.01 [503.89, 878.88]	722.68 [495.80, 992.45]	802.61 [677.35, 986.40]	0.223
ATR1 (ng/mL)	91.84 [22.72, 1350.96]	138.89 [25.74, 221.86]	189.42 [11.62, 1438.19]	97.71 [11.17, 868.94]	0.826
ROUTINE LABORATORY PARAMETERS					
Hemoglobin (g/dL)	14.25 [12.78, 14.65]	14.20 [13.75, 15.20]	13.50 [12.80, 14.50]	14.05 [13.03, 14.85]	0.128
Hematocrit (%)	41.55 [37.83, 43.90]	41.80 [40.35, 44.60]	41.00 [39.10, 42.60]	41.60 [39.93, 43.88]	0.606
MCV (fl)	89.25 [87.68, 90.30]	89.80 [87.85, 91.85]	89.80 [88.60, 91.20]	89.25 [86.53, 91.65]	0.895
MCH (pg)	29.50 [29.23, 30.38]	30.60 [29.60, 31.45]	29.90 [28.70, 30.20]	30.20 [29.23, 30.78]	0.120
Platelets (G/L)	261.00 [203.50, 297.25]	275.00 [241.00, 288.50]	243.00 [213.00, 285.00]	252.50 [225.00, 300.00]	0.498
Leucocytes (G/L)	6.54 [5.80, 7.64]	6.41 [5.21, 7.13]	6.15 [5.49, 6.62]	6.16 [5.45, 7.00]	0.468
NT-proBNP (pg/mL)	41.15 [32.43, 68.68]	49.80 [29.80, 66.20]	58.00 [32.90, 80.70]	44.20 [29.83, 104.25]	0.957
hs-CRP (mg/dL)	0.08 [0.06, 0.23]	0.04 [0.03, 0.08]	0.09 [0.04, 0.25]	0.09 [0.05, 0.19]	0.234
Interleukin-6 (pg/mL)	1.80 [1.50, 2.18]	1.50 [1.50, 1.72]	1.50 [1.50, 2.53]	1.63 [1.50, 2.49]	0.478
IgG (mg/dL)	1155.00 [1062.50, 1347.50]	1050.00 [898.00, 1240.00]	1115.00 [930.25, 1217.50]	1040.00 [972.00, 1215.00]	0.157
IgA (mg/dL)	189.00 [132.25, 284.25]	198.00 [169.50, 248.00]	200.00 [153.75, 252.75]	166.00 [121.00, 212.00]	0.160
IgM (mg/dL)	106.50 [73.83, 146.00]	102.00 [64.55, 144.00]	102.00 [88.70, 140.00]	103.00 [65.00, 161.00]	0.867
IgE (kIU/L)	41.10 [17.20, 77.20]	25.50 [12.68, 75.85]	24.60 [8.08, 45.30]	26.70 [11.30, 47.20]	0.626
IgGsubclass1 (mg/dL)	709.50 [629.50, 836.00]	673.50 [573.50, 776.00]	696.00 [562.00, 794.00]	674.50 [593.25, 747.25]	0.719
IgGsubclass2 (mg/dL)	377.50 [278.50, 444.00]	289.00 [232.75, 344.25]	335.00 [266.00, 410.00]	325.00 [264.75, 424.50]	0.281
IgGsubclass3 (mg/dL)	35.05 [23.35, 43.08]	33.85 [22.78, 41.18]	28.30 [22.60, 39.50]	33.80 [28.50, 46.40]	0.536
IgGsubclass4 (mg/dL)	61.70 [27.25, 89.25]	25.10 [13.78, 71.50]	53.50 [34.10, 98.00]	38.20 [19.33, 95.43]	0.524
ECHO PARAMETERS					
Left ventricle size (mm)	42.50 [40.25, 47.00]	44.00 [40.00, 46.00]	41.00 [40.00, 43.00]	43.00 [40.50, 46.50]	0.400
Right ventricle size (mm)	29.50 [26.00, 33.00]	29.00 [28.00, 31.75]	28.50 [25.25, 30.00]	28.00 [26.00, 33.00]	0.569
Right atrium size (mm)	45.50 [41.50, 49.50]	44.00 [42.75, 46.50]	44.50 [40.00, 45.75]	45.00 [42.00, 49.50]	0.683

ACEi = angiotensin converting enzyme inhibitor, Ang1-7 = Angiotensin (1-7), ARB = angiotensin receptor blocker, ATR1 = Angiotensin II receptor 1, BAU = binding antibody units, Beta-2 AR = Beta-2, BMI = body mass index, ETAR = endothelin receptor type A, hs-CRP = high-sensitivity C-reactive protein, mAChR3 = muscarinic acetylcholine receptor 3, SpikeAB = Spike Protein Antibody; ^1^ Median [IQR]; *n* (%); ^2^ Kruskal-Wallis rank sum test; Pearson’s Chi-squared test. Bold numbers indicate significant *p* Values.

**Table 3:** Overview of EQ-5D-3L responses.

EQ5D Item	Orthostatic Intolerance *n* = 38 (Available Questionnaires *n* = 19)	No Orthostatic Intolerance *n* = 62 (Available Questionnaires *n* = 41)	*p*-Value
**Mobility:** **walking about**			0.896
No problems	11 (58%)	23 (56%)	
Some problems	8 (42%)	18 (44%)	
Unable to	0	0	
**Self-care:** **washing and getting dressed**			0.707
No problems	17 (89%)	34 (83%)	
Some problems	2 (11%)	6 (15%)	
Unable to	0	1 (2.4%)	
Usual activities *			0.468
No problems	5 (26%)	6 (15%)	
Some problems	12 (63%)	32 (78%)	
Unable to	2 (11%)	3 (7.3%)	
**Pain/discomfort**			0.143
None	1 (5.3%)	4 (9.8%)	
Moderate	17 (89%)	27 (66%)	
Extreme	1 (5.3%)	10 (24%)	
**Anxiety/depression**			0.682
None	8 (42%)	16 (39%)	
Moderate	9 (47%)	17 (41%)	
Extreme	2 (11%)	8 (20%)	

* (e.g., work, study, housework, family or leisure activities). EQ-5D = quality of life questionnaire.

**Table 4:** Spearman r between ETAR autoantibody concentration and EQ-5D scores.

EQ-5D Item	Spearman r	95% Confidence Interval	*p* Value
EQ-5D Mobility	0.22	−0.04 to 0.46	0.0861
EQ-5D Self-care	−0.10	−0.35 to 0.16	0.4376
EQ-5D Usual Activities	0.33	0.08 to 0.54	**0.0099**
EQ-5D Pain/Discomfort	0.17	−0.09 to 0.42	0.1810
EQ-5D Anxiety/Depression	−0.04	−0.29 to 0.23	0.7843

EQ-5D = quality of life questionnaire, ETAR = endothelin receptor type A. Bold text indicates significant *p* Value.

## Data Availability

Data used in this study are available from the corresponding author upon reasonable request.

## Supplementary Materials

The following supporting information can be downloaded at https://www.mdpi.com/article/10.3390/biom15020300/s1: Table S1: Baseline data of long COVID patients and control groups. Table S2: Patient characteristics of long COVID cohort stratified by gender. Figure S1: Concentration of antibodies against endothelin A receptor (ETAR) in long COVID patients and control groups. Figure S2: Correlation matrix of biomarkers measured by enzyme-linked immunosorbent assay. Figure S3: Correlation between ETAR autoantibody concentration and antispike protein concentration in long COVID patients, stratified by orthostatic intolerance or no orthostatic intolerance. Figure S4: Correlation matrices of routine laboratory parameters and autoantibodies against vasoactive peptides.
