# Supplementary material for: Circulating Autoantibodies Against Vasoactive Biomarkers Related to Orthostatic Intolerance in Long COVID Patients Compared to No-Long-COVID Populations: A Case-Control Study
Source: Biomolecules. 2025 Feb 18;15(2):300. doi: 10.3390/biom15020300 (PMC11853648; doi:10.3390/biom15020300)
Supplement: Supplementary file 1 [file biomolecules-15-00300-s001.zip › biomolecules-3364056-supplementary.pdf]

Supplementary materials

Title: Circulating autoantibodies against vasoactive biomarkers related to orthostatic intolerance in long COVID patients compared to no-long-COVID populations: a case-control study.

Authors: Emilie Han, Katrin Müller-Zlabinger, Ena Hasimbegovic, Laura Poschenreithner, Nina Kastner, Babette Maleiner, Kevin Hamzaraj, Andreas Spannbauer, Martin Riesenhuber, Anja Vavrikova, Antonia Domanig, Christian Nitsche, Dominika Lukovic, Thomas A. Zelniker, Mariann Gyöngyösi

Table of contents

Number of Supplementary Tables: 2
Supplementary Table S1. Baseline data of long COVID patients and control groups.
Supplementary Table S2. Patient characteristics of long COVID cohort stratified by gender.

Number of Supplementary Figures: 4
Supplementary Figure S1. Concentration of antibodies against endothelin A receptor (ETAR) in long COVID patients and control groups.
Supplementary Figure S2. Correlation matrix of biomarkers measured by enzyme-linked immunosorbent assay.
Supplementary Figure S3. Correlation between ETAR autoantibody concentration and antispike protein concentration in long COVID patients, stratified by orthostatic intolerance or no orthostatic intolerance.
Supplementary Figure S4. Correlation matrices of routine laboratory parameters and autoantibodies against vasoactive peptides.

Supplementary Table S1. Baseline data of long COVID patients and control groups.

Characteristic	Total	Long-COVID	Asymptomatic post-COVID controls	Vaccinated controls	Healthy controls
**N**	**160**	**100**	**20**	**20**	**20**
Age (year), mean (SD)	43.8 (12.4)	41.7 (12.7)	44.8 (12.0)	52.7 (7.1)	44.5 (12.0)
Female, n (%)	118 (73.8%)	74 (74.0%)	14 (70.0%)	17 (85.0%)	13 (65.0%)
Vasoactive peptides					
Beta-2 AR (U/L)	13.4 [7.1, 24.3]	13.6 [9.2, 26.1]+	14.7 [6.4, 43.5]+	8.1 [5.4, 28.3]	11.1 [4.1, 19.4]
Beta-2 AR elevated (> 14 U/L)	114 (71%)	78 (78%)	12 (60%)	10 (50%)	14 (70%)
ETAR (U/L)	17.6 [11.9, 36.4]	16.5 [12.2, 40.4]*	23.2 [18.2, 33.7]*	22.1 [16.7, 44.0]*	10.1 [7.2, 12.6]
ETAR elevated (>17 U/L)	142 (89%)	92 (92%)	20 (100%)	19 (95%)	11 (55%)
mAChR3 (U/L)	14.7 [9.9, 30.9]	15.6 [10.5, 31.0]	16.5 [11.8, 37.4]	13.4 [9.1, 26.0]	14.4 [7.9, 23.2]
mAChR3 elevated (>10 U/L)	119 (74%)	77 (77%)	17 (85%)	13 (65%)	12 (60%)
Ang1-7 (pg/mL)	760.1 [532.8, 963.0]	791 [564, 967]#	847 [543, 1040]	715 [653, 892]	414 [183, 932]
ATR1 (ng/mL)	126.1 [4.2, 1074.0]	76.8 [0.3, 239]	136 [13.8, 942]	0.65 [0.0, 295]	30.8 [1.6, 131]

Beta-2 AR=Beta-2 adrenergic receptor, ETAR=endothelin receptor type A, mAChR3= muscarinic acetylcholine receptor 3, Ang1-7=Angiotensin (1-7), ATR1= Angiotensin II receptor 1. Continuous data displayed as median [Q1, Q3] if not otherwise specified. Categorical data displayed as n (%).
*p<0.05 between Long-COVID/Asymptomatic post-COVID controls/Vaccinated controls vs Healthy controls
+p=0.063 (ANOVA) between Long-COVID vs Vaccinated/Healthy controls
#p=0.074 (ANOVA) between Long-COVID vs Healthy controls

Supplementary Table S2. Patient characteristics of long COVID cohort stratified by gender.

	Total (N=100)	Women (N=74)	Men (N=26)
**Age (year),** mean (SD)	41.7 (12.7)	41.7 (12.9)	41.7 (12.6)
**Body mass index** (kg/m^2)	23.8 [20.7, 26.8]	23.0 [20.5, 25.9]	25.3 [21.6, 28.0]
**Hypertension**	29 (29.0%)	20 (27.0%)	9 (34.6%)
**Diabetes mellitus**	0 (0.0%)	0 (0.0%)	0 (0.0%)
**Hyperlipidemia**	17 (17.0%)	11 (14.9%)	6 (23.1%)
**Current smoker**	11 (15.5%)	8 (14.5%)	3 (18.8%)
**Time between COVID-19 positivity and first clinical presentation (days)**	222.5 [160.3, 315.3]	226.0 [155.0, 314.0]	214.0 [169.8, 313.5]
**COVID vaccinated at baseline**	48 (48.0%)	33 (44.6%)	15 (57.7%)
**Time between COVID vaccine and first clinical presentation (days)**	95.5 [26.8, 154.5]	96.0 [23.0, 149.0]	95.0 [64.0, 166.0]
**Systolic blood pressure** (mmHg)	130.0 [120.0, 140.0]	125.0 [120.0, 140.0]	130.0 [130.0, 140.0]
**Diastolic blood pressure** (mmHg)	85.0 [80.0, 90.0]	80.0 [80.0, 90.0]	90.0 [80.0, 90.0]
**Heart rate,** (bpm) mean (SD)	71.9 (10.5)	73.4 (9.5)	66.7 (11.6)
**CONCOMITANT MEDICATION**			
**Betablocker**	10 (10.0%)	6 (8.1%)	4 (15.4%)
**RAS-inhibitor (ACEi, ARB)**	12 (12.0%)	6 (8.1%)	6 (23.0%)
**Lipid lowering therapy**	9 (9.0%)	6 (8.1%)	3 (11.5%)
**LAB VALUES**			
**Hemoglobin (g/dL)**	13.9 [13.1, 14.7]	13.4 [12.8, 14.2]	14.9 [14.5, 15.5]
**Platelets (G/L)**	254.5 [215.5, 290.5]	263.0 [231.2, 303.5]	224.5 [197.0, 261.8]
**Leucocytes (G/L)**	6.3 [5.5, 7.1]	6.4 [5.8, 7.2]	5.6 [4.7, 7.0]
**hs-CRP (mg/dL)**	0.06 [0.05, 0.16]	0.06 [0.04, 0.14]	0.06 [0.05, 0.20]
**IL-6 (pg/mL)**	1.54 [1.50, 2.23]	1.70 [1.50, 2.34]	1.50 [1.50, 2.02]
**Procalcitonin (ng/mL)**	0.03 [0.02, 0.04]	0.03 [0.02, 0.03]	0.03 [0.02, 0.04]
**NT-proBNP (pg/mL)**	46.5 [31.0, 80.8]	55.0 [34.2, 84.6]	30.6 [17.8, 45.5]
**IgG (mg/dL)**	1110.0 [970.5, 1230.0]	1140.0 [973.5, 1292.5]	1060.0 [939.5, 1157.5]
**IgA (mg/dL)**	187.5 [140.2, 232.8]	180.5 [139.5, 227.5]	206.0 [152.8, 259.5]
**IgM (mg/dL)**	102.0 [71.6, 144.0]	109.0 [81.1, 161.0]	75.4 [63.5, 115.0]
**IgE (kIU/L)**	30.9 [11.0, 58.8]	37.0 [20.4, 78.0]	7.6 [4.7, 34.0]
**IgGsubclass1 (mg/dL)**	691.0 [599.0, 783.0]	704.5 [604.2, 794.8]	679.0 [566.0, 749.0]
**IgGsubclass2 (mg/dL)**	333.0 [265.0, 429.0]	345.0 [272.0, 438.2]	287.0 [254.0, 375.0]
**IgGsubclass3 (mg/dL)**	33.5 [23.4, 43.2]	33.6 [25.1, 41.9]	33.2 [21.9, 45.2]
**IgGsubclass4 (mg/dL)**	53.4 [23.6, 90.4]	45.6 [24.4, 97.2]	54.1 [16.4, 75.2]
**Beta-2 AR (U/L)**	13.6 [9.2, 26.1]	13.9 [9.4, 26.5]	13.6 [8.4, 22.7]
**ETAR (U/L)**	16.5 [12.2, 40.4]	16.4 [12.5, 39.0]	18.7 [11.9, 41.1]
**mAChR3 (U/L)**	15.6 [10.5, 31.0]	16.3 [10.5, 31.6]	14.2 [10.3, 27.3]

**Ang1-7 (pg/mL)**	790.6 [563.4, 965.3]	818.7 [564.0, 961.0]	780.2 [544.7, 999.3]
**ATR1 (ng/mL)**	126.04 [11.5, 1042.8]	142.0 [5.6, 1019.5]	93.5 [38.0, 1227.8]
**ECHO PARAMETERS**			
**Left ventricle size (mm)**	42.0 [40.0, 45.5]	41.0 [40.0, 43.0]	45.5 [42.3, 49.8]
**Right ventricle size (mm)**	28.0 [26.0, 31.5]	27.0 [26.0, 30.0]	31.5 [28.0, 37.5]
**Right atrium size (mm)**	45.0 [41.0, 48.0]	43.5 [40.0, 46.0]	49.0 [44.3, 54.0]

Continuous data displayed as median [Q1, Q3] if not otherwise specified. Categorical data displayed as counts (n) and column percentages (%).
ACEi=angiotensin converting enzyme inhibitor, Ang1-7=Angiotensin (1-7), ARB=angiotensin receptor blocker, ATR1= Angiotensin II receptor 1, Beta-2 AR=Beta-2 adrenergic receptor, ETAR=endothelin receptor type A, hs-CRP=high-sensitvity C-reactive protein, mAChR3= muscarinic acetylcholine receptor 3.

Supplementary Figure S1. Concentration of antibodies against endothelin A receptor (ETAR) in long COVID patients and control groups.

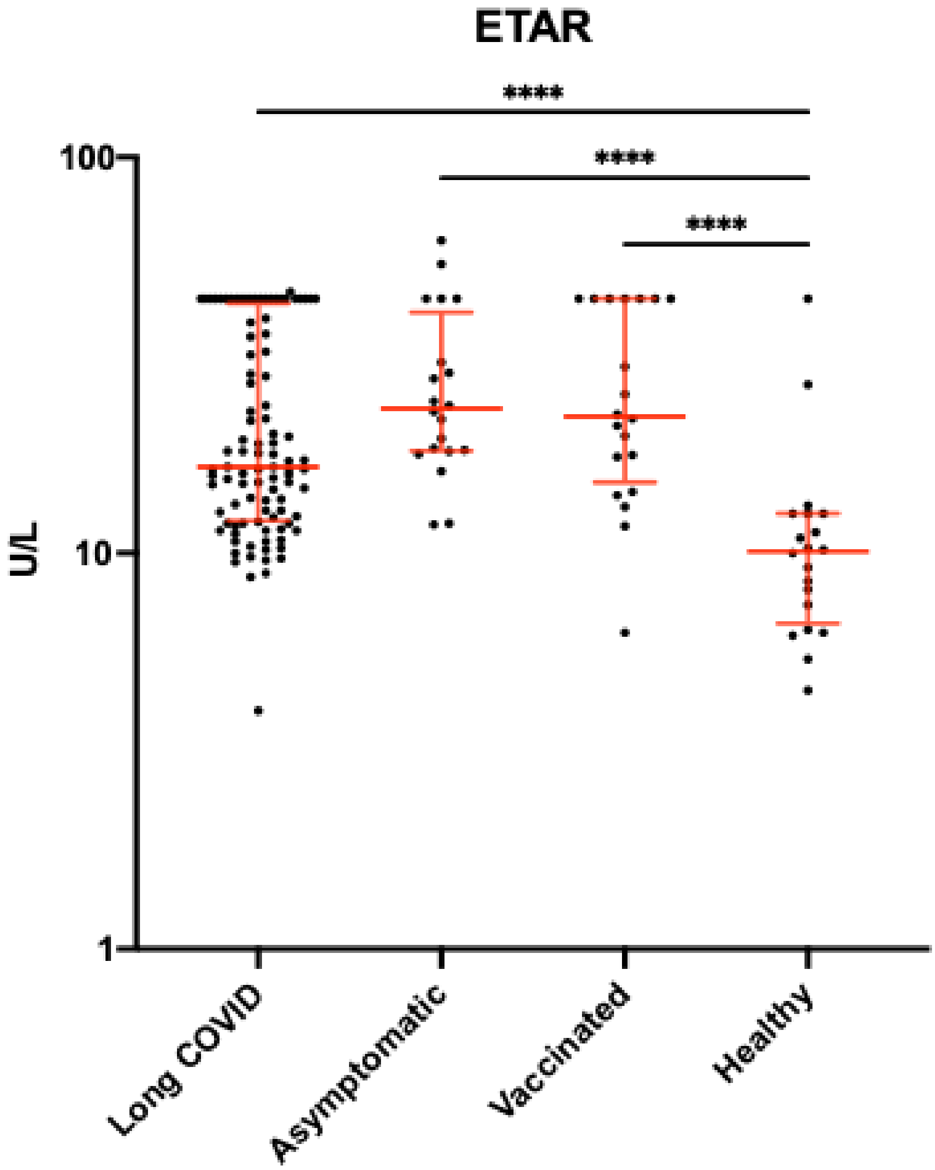

ETAR = endothelin A receptor, asymptomatic = after COVID-19 infection without long COVID, vaccinated = received COVID-19 vaccination but without infection, healthy = completely naïve towards the COVID-19 antispike protein and negative for nucleocapsid antigen with no clinical history of COVID-19 symptoms.
Median and IQR (as error bars) are shown in red.

Supplementary Figure S2. A. Correlation matrix of biomarkers measured by enzyme-linked immunosorbent assay. The correlation coefficients with an asterisk (*) are statistically significant. **B.** Scatter plot of autoantibody concentrations of ETAR and mAChR3. **C.** Scatter plot of autoantibody concentrations of ETAR and beta-2 AR. **D.** Scatter plot of autoantibody concentrations of ETAR and Ang1-7. **E.** Scatter plot of autoantibody concentrations of ETAR and ATR1.

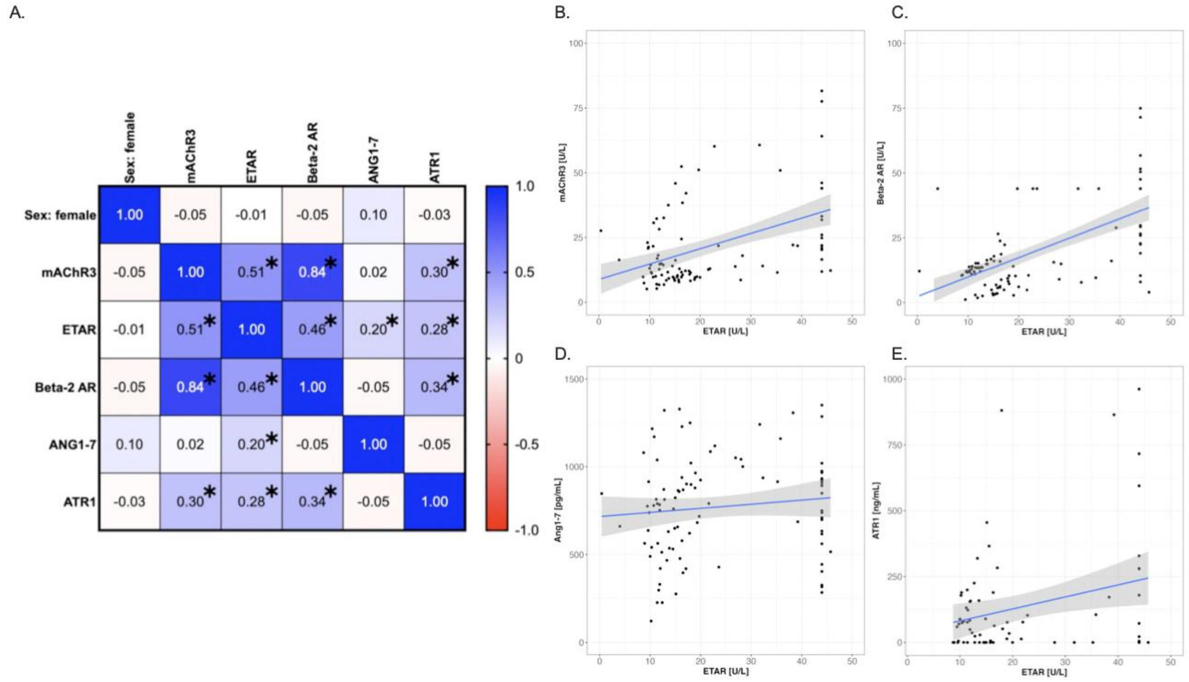

Beta-2 AR=Beta-2 adrenergic receptor, ETAR=endothelin receptor type A, mAChR3= muscarinic acetylcholine receptor 3, Ang1-7=Angiotensin (1-7), ATR1= Angiotensin II receptor 1.

Supplementary Figure S3. Correlation between ETAR autoantibody concentration and antispike protein concentration in long COVID patients, stratified by orthostatic intolerance or no orthostatic intolerance. **A.** Scatter plot of ETAR autoantibody and antispike protein for all long COVID patients; **B.** only for long COVID patients with orthostatic intolerance; **C.** only for long COVID patients without orthostatic intolerance.

A.

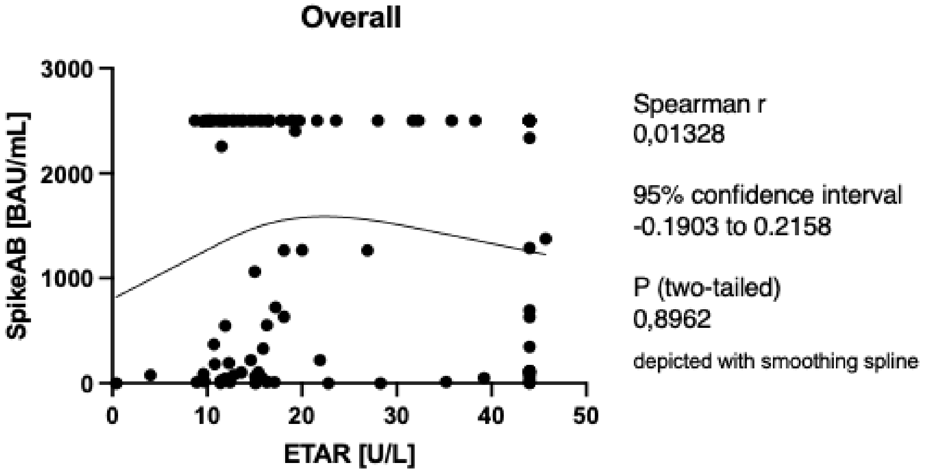

B.

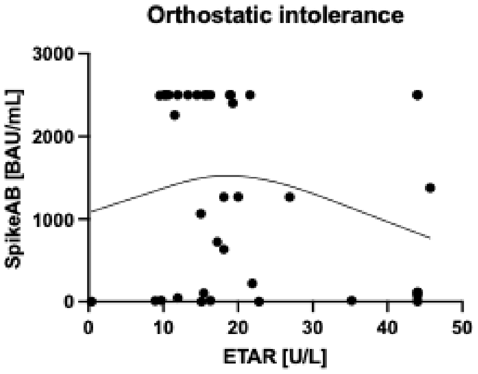

C.

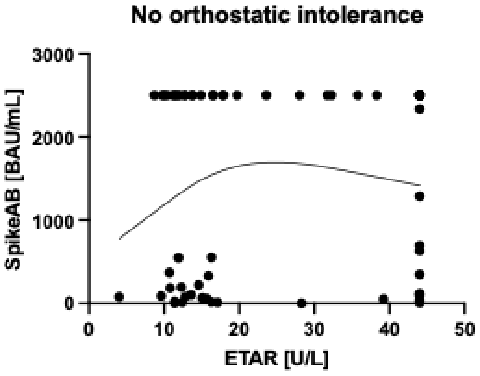

ETAR = endothelin receptor type A autoantibody concentration, SpikeAB = spike antibody.

Supplementary Figure S4. Correlation matrices of routine laboratory parameters and autoantibodies against vasoactive peptides.

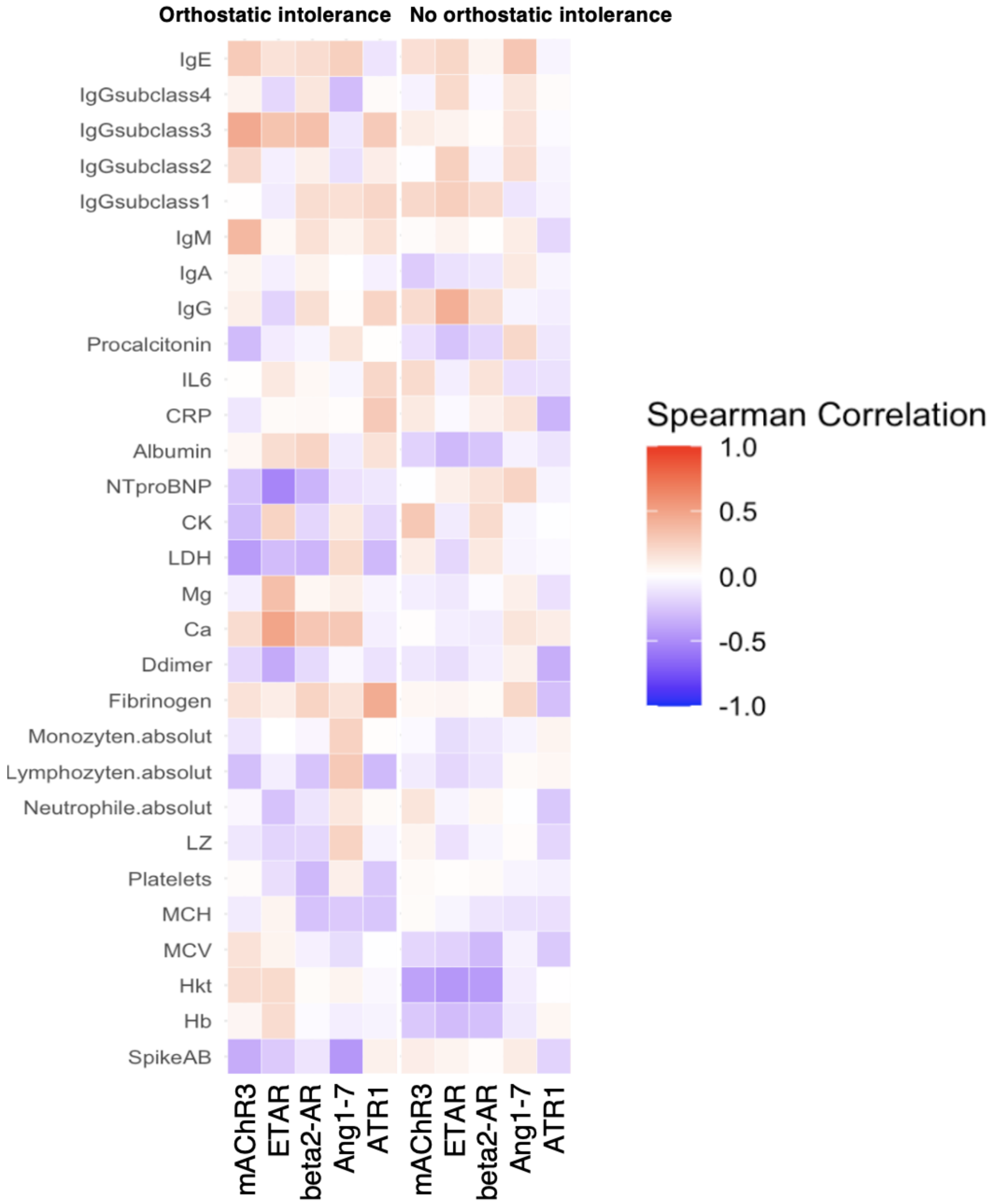

AB=antibody, Ang1-7=Angiotensin (1-7), ATR1= Angiotensin II receptor 1, Beta-2 AR=Beta-2 adrenergic receptor, ETAR=endothelin receptor type A, CRP=high-sensitvity C-reactive protein, Hb = hemoglobin, LZ = leucocytes, mAChR3= muscarinic acetylcholine receptor 3.
